# Bio-Based Materials as a Sustainable Solution for the Remediation of Contaminated Marine Sediments: An LCA Case Study

**DOI:** 10.3390/polym16152101

**Published:** 2024-07-23

**Authors:** Milvia Elena Di Clemente, George Barjoveanu, Francesco Todaro, Michele Notarnicola, Carmen Teodosiu

**Affiliations:** 1 Department of Civil, Environmental, Land, Building Engineering and Chemistry (DICATECh), Polytechnic University of Bari, 70125 Bari, Italyfrancesco.todaro@poliba.it (F.T.); michele.notarnicola@poliba.it (M.N.); 2 Department of Environmental Engineering and Management, “Cristofor Simionescu” Faculty of Chemical Engineering and Environmental Protection, “Gheorghe Asachi” Technical University of Iasi, 700050 Iasi, Romania

**Keywords:** cellulose-based adsorbents, organic pollutants, heavy metals, marine sediments, life cycle assessment

## Abstract

Contaminated sediments may induce long-term risks to humans and ecosystems due to the accumulation of priority and emerging inorganic and organic pollutants having toxic and bio-accumulation properties that could become a secondary pollution source. This study focused on the screening of novel bio-based materials to be used in the decontamination of marine sediments considering technical and environmental criteria. It aimed to compare the environmental impacts of cellulose-based adsorbents produced at lab scale by using different syntheses protocols that involved cellulose functionalization by oxidation and branching, followed by structuring of an aerogel-like material via Soxhlet extraction and freeze-drying or their combination. As model pollutants, we used 4-nitrobenzaldehyde, 4-nitrophenol, methylene blue, and two heavy metals, i.e., cadmium and chromium. When comparing the three materials obtained by only employing the Soxhlet extractor with different solvents (without freeze-dying), it was observed that the material obtained with methanol did not have a good structure and was rigid and more compact than the others. A Life Cycle Assessment (LCA) was conducted to evaluate the environmental performance of the novel materials. Apart from the hierarchical categorization of the materials based on their technical and environmental performance in eliminating organic pollutants and heavy metal ions, it was demonstrated that the cellulose-based material obtained via Soxhlet extraction with ethanol was a better choice, since it had lower environmental impacts and highest adsorption capacity for the model pollutants. LCA is a useful tool to optimize the sustainability of sorbent materials alongside lab-scale experiments and confirms that the right direction to produce new performant and sustainable adsorbent materials involves not only choosing wastes as starting materials, but also optimizing the consumption of electricity used for the production processes. The main results also highlight the need for precise data in LCA studies based on lab-scale processes and the potential for small-scale optimization to reduce the environmental impacts.

## 1. Introduction

An important environmental problem of this century is seawater quality and its sediment pollution. The development of coastal human activities, such as agriculture, urbanization, and industry, during the last century has led to the release of many hazardous chemicals in the aquatic environment. Aquatic sediments have been identified as the ultimate receptor for many of these pollutants, and sedimental resuspension leads contaminants to re-enter the overlying water, thus making them available for benthic organisms and subsequently re-enter aquatic food chains [1].

Because of these risks, the remediation of contaminated marine sediments is currently one of the most challenging problems in the environmental protection area. The two major techniques used for marine sediment remediation are ex situ solidification/stabilization and in situ reactive capping (ISC). Both are efficient at neutralizing and/or degrading contaminants from marine sediments, but the ex situ solidification/stabilization technique, even if it greatly diminishes the potential risks associated with metals leaching, induces additional impacts in various other areas that are associated with the use of stabilizers and solidification agents [2].

ISC stands out as a reliable technology that consists of placing a clean material layer (clean sediment, sand, or a combination of materials) on top of contaminated sediments with the following purposes: (a) separate the contaminated sediment area from the benthic environment; (b) prevent the resuspension and transport of contaminated sediments; (c) reduce the dissolved contaminant fluxes into the overlying water column. Both field and bench-scale capping are presented in the literature [3,4,5] to demonstrate how ISC controls contamination in sediments and in the overlying water.

In situ reactive capping uses active materials such as activated carbon, zero-valent iron, or organoclay to obtain both chemical isolation and degradation with an efficiency varying between 40% and 95%. As demonstrated by an LCA analysis, it is possible to state that ISC is an effective remediation strategy with small impacts and low costs [6].

The main advantages of ISC-based remediation are the lower costs compared to those of other sediment remediation technologies (e.g., ex situ remedies) [7], its suitability for sequestering both organic and inorganic pollutants [8], and the advantages of being an in situ technique, such as the reduction of resuspension phenomena [9].

Adequate materials have been developed and implemented to block or degrade pollutants in marine sediments and to prevent their resuspension into the overlying water [10]. In the last two decades, the most used materials were activated carbon, apatite, biochar, coke, organoclays, zeolites, and zero-valent iron [11]. Many studies were carried out on the actual effectiveness of these materials, but nowadays, the focus is on materials of nanometric size. Nanomaterials are materials with sizes in the nano-scale (1–100 nm) in at least one dimension and include nano-objects and nanoparticles [12,13]. These materials are appreciated due to their excellent adsorption and catalytic properties, large specific surface areas, associated sorption sites, lower temperature modification, shorter interparticle diffusion distance, more tunable pore size, and different surface chemistry with respect to other materials [14]. Moreover, the use of nanomaterials for marine sediments remediation is more effective compared to that of conventional remediation, since these materials show a high surface-area-to-volume ratio, high reactivity, and a target-specific ability to capture toxic compounds [15], making the extension of in situ remediation possible. The main characteristics required for in situ remediation are the following: (i) a high reactivity for pollutants’ removal; (ii) enough mobility in the presence of porous media; (iii) sufficient reactive longevity; (iv) low toxicity [16].

All the studies conducted on nanomaterials demonstrated that they are effective in stabilizing/adsorbing heavy metal(loid)s, thereby decreasing their mobility and bioavailability. Unfortunately, heavy metals cannot be degraded into non-toxic substances in sediments, but can be immobilized/passivized by adding different amendments to the sediments. The most interesting and effective materials for this purpose were found to be nano-zero-valent iron (nZVI) [16], nanohydroxyapatite [17], carbon nanotubes [18], electrospun nanofibers [19], and nanostructured cellulose sponges [20,21,22]. These materials basically exploit two principles: the difference in surface charges and, thus, the ability to attract and retain contaminants thanks to the electrostatic charges present on the surface (nZVI and carbon nanotubes), and surface functional groups such as -COOH and/or -NH that, by being highly reactive, bind contaminants and block them (nanoapatite, electrospun fibers, and nanostructured cellulose). One common problem with these materials is that, although they are considered good adsorbent materials, some of them could be expensive and not able to retain all contaminant types [23,24]. On the other hand, cellulose-based nanostructured sponges demonstrated effectiveness in blocking contaminants (heavy metals) and degrading organics, which is due to the presence of various superficial functional groups on their outer surface (mainly carboxyl and ammino) [20,25,26,27].

The cellulose-based nanostructured sponge (CNS) is an innovative material that has cellulose as a starting material, which can be also obtained from wastes such as agricultural residues, cotton, food waste, municipal and industrial biowaste such as used paper, carton, and wood from demolition sites. Appropriate branching agents are then added to cellulose, which gives it a nano-sized sponge-like structure. The synthesis process consists of two steps: cellulose oxidation and the addition of a branching agent. This material has a high specific surface area, a porous structure, good mechanical stability in water, and shape-memory capability [21,28]. Its effectiveness in blocking heavy metal ions and degrading organic contaminants is due to the presence of various functional groups on its outer surface. These groups are derived from the used branching agents, typically, branched (poly)ethyleneimine (b-PEI) and carboxylates [21,26,29,30,31].

Life Cycle Assessment (LCA) is an important tool for evaluating the environmental impact and carbon footprint that the synthesis processes and implementation of novel materials and technologies have. In the last decade, LCA was applied to marine sediment remediation as a valuable tool for assessing the environmental footprint of sediment remediation projects and for sustainable sediment management [2,6,32].

Apart from the technical performance of these materials, an important aspect is to assess their environmental performance, because there can be multiple syntheses routes for the same material, and LCA could provide support to find the more sustainable one [33]. Also, LCA is useful in identifying the most important contributing processes or fluxes to a specific impact category (e.g., the major contributions might be related to electricity consumption or the use of input chemicals) [34]. Using LCA to evaluate laboratory-scale synthesis protocols is more complicated than using it for large-scale applications due to the high uncertainties related to data representativity and process specificity [25]; however, LCA remains a useful tool for identifying the critical steps in product development as well as for scaling up production. It was found that certain materials had a significantly higher environmental impact when produced in small quantities at laboratory scale than when produced on a large scale [35,36]. Although nanomaterials are a promising resource for the remediation of heavy metal(loid)-polluted sediments, few studies have been published on immobilizing/adsorbing heavy metal(loid)s by utilizing nanomaterials for marine sediment remediation.

This study’s research objectives were the following: (a) screening cellulose bio-based materials to be used in the decontamination of marine sediments, considering their technical and environmental performances in eliminating organic pollutants and heavy metal ions; (b) considering two functional units for the environmental performance evaluation by LCA, i.e., 1 g of produced nanocellulose bio-based material and 1 mg of sorbed pollutant taking into account the material functionality, as ISO 14040 recommends; (c) provide a sensitivity analysis to understand and evaluate the stochastic uncertainties related to the mode in which inventory data affect the life cycle impact results.

To the best of our knowledge, these types of cellulose-based materials used as pollutant sorbents have not been previously evaluated by means of LCA from an environmental sustainability point of view. A previous study [35] used LCA to just evaluate the impact of the freeze-dry process used to modify cellulose. In this study, we used LCA to compare multiple syntheses routes to obtain and test these promising materials. It was important to conduct this early-stage environmental analysis by LCA to verify if the material development and subsequent production are sustainable by taking into consideration the material functionality from the use phase.

## 2. Materials and Methods

### 2.1. Material Synthesis and Characterization

**Figure 1:**
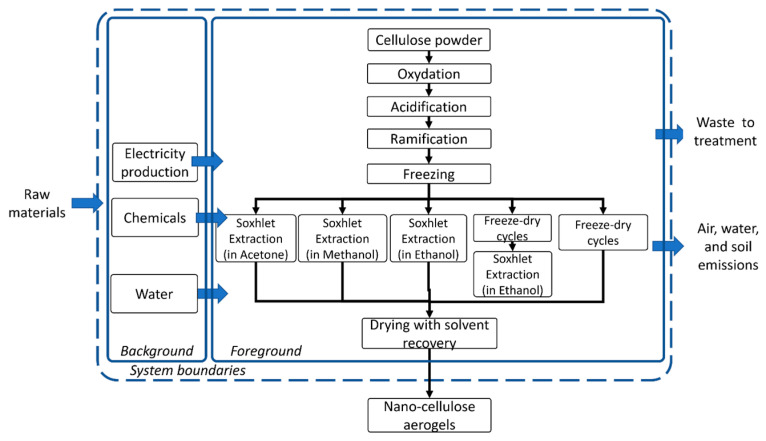
Nanocellulose production routes and system limits.

To obtain the desired functionalized bio-composite, the synthesis protocol, adopted in our laboratory as reported by [28] and presented in Figure 1, involved the oxidation of cellulose microcrystalline powder (Sigma-Aldrich, Milan, Italy) with 2,2,6,6-tetramethylpiperidinyloxyl (TEMPO) (>97.5%, Sigma-Aldrich, Milan, Italy) and KBr (ACS reagent, ≥99.0%, Sigma-Aldrich, Milan, Italy), acidified and branched with branched polyethyleneimine (bPEI), (average Mw ~25,000 by LS, Sigma Aldrich, Milan, Italy) and frozen overnight. Cellulose oxidation refers to the conversion of the C6 hydroxyl group to a salt C6 carboxylate group via an intermediary aldehyde [37]. The final goal of the synthesis is to obtain a stable aerogel that would present the necessary structural and morphological characteristics to be used as a sorbent for various pollutants. Two methods were tested and compared for this purpose: the use of Soxhlet extraction with few solvents tested and the freeze-drying method.

Freeze-drying is a process that allows for the removal of water from an organic substance with the least possible deterioration of the substance’s structure and components. Its principle involves the application of heat to a frozen and vacuumed material, with the water in the sample segregating as ice crystals then being extracted directly as vapor by sublimation. The pressure values are below 6.10 mbar, which corresponds to the triple point of water, i.e., the conditions that allow for the simultaneous presence of water in the three solid, liquid, and vapor phases, which makes it possible to directly separate the water vapor and to maintain the solid phase in its initial structural and morphological shape [38]. The extracted water vapor must be captured by freezing in condensers, the incondensable gases being extracted and removed by a vacuum pump. The process is conducted under carefully controlled temperature and pressure conditions to avoid damages to the material structure, so that the original matrix is almost perfectly restorable when rehydration is desired at the time of use [39]. The process involved freezing the samples with liquid nitrogen and placing them in the freeze-dryer for 48 h. Next, the samples were dried in an oven for about 16 h at 80 °C and then washed for 24 h with ethanol in a Soxhlet extractor to remove all the excess bPEI [20]. The whole procedure took a total time of about 90 h.

In the Soxhlet extraction process, the solvent is heated, and the solvent vapors rise along a distillation arm and trickle into the chamber that houses the thimble full of solid. The condenser ensures that the solvent vapor cools and drips back into the chamber that houses the solid material. The chamber containing the solid material slowly fills with hot solvent that will dissolve the desired part of the compound. When the Soxhlet chamber is nearly full, it is emptied by the siphon, and the solvent is returned to the distillation flask. This cycle can be repeated several times, for hours [40].

During each cycle, some of the non-volatile compound dissolves in the solvent. After many cycles, the desired compound is concentrated in the distillation flask; in this case, water and the bPEI excess were removed from the sample. The advantage of this system is that instead of passing many portions of hot solvent through the sample, only one batch of solvent is recycled.

In our case, the non-soluble part of the extracted solid that remained in the thimble was the nanocellulose aerogel. The sample thus obtained was thermally treated in a vacuum oven at 60 °C for about 2 h. The choice of the extraction solvent was investigated, and the solvents used were acetone, methanol, and ethanol [41].

The three model organic pollutants for the adsorption tests were 4-nitrophenol (NP), a priority pollutant (ReagentPlus^®^, ≥99%, Sigma-Aldrich, Milan, Italy), 4-nitrobenzaldehyde (NBA) (GC, 98%, Sigma-Aldrich, Milan, Italy), and methylene blue (MB) (Sigma-Aldrich, Milan, Italy), while the inorganic contaminants were heavy metal ions (Cd^2+^ and Cr^2+^), (ACS Reagents, 99%, Sigma-Aldrich, Milan, Italy) [42,43]. In total, 10 mL of the dye solution and 100 mg of adsorbent material were mixed under stirring for 24 h (batch tests). The solution was analyzed with a UV-Vis spectrometer (JASCO V670, Jasco Corporation, Tokio, Japan) to observe the decreasing concentration of the dye related to the adsorption on the bio-based material. To study the kinetic behavior, the solution was analyzed after 15 min, 30 min, 1 h, 2 h, 3 h, 5 h, 7 h, and finally 24 h. Four materials were tested: a cellulose-based material obtained by freeze-drying (NC-FD), a cellulose-based material obtained by freeze-drying and Soxhlet extraction with ethanol (≥99.8%, EMSURE^®^, Merck-Millipore, Milan, Italy), NC-FD-SOX), a cellulose-based material obtained by Soxhlet extraction with acetone (ACS reagent, ≥99.5%, Sigma-Aldrich, Milan, Italy), (NC-SOX-C3H4O), and a cellulose-based material obtained by Soxhlet extraction with ethanol (NC-SOX-ETH). The sorption performances for these materials of the 3 organic pollutants and the 2 heavy metal ions, presented in Table 1, show that the material that was obtained with the Soxhlet extractor using ethanol as a solvent had the best sorption capacity for organics. The two materials obtained by the freeze-dry processes were the worst, because one of them (NC-FD) was very fragile under stirring and fell apart in solution during the 24 h sorption process; so, it was impossible to measure 4-nitrobenzadehyde concentration. For the other freeze-dried material (NC-FD-SOX), the UV-VIS signal was out of scale after 30 min, probably because of some wash-out contamination from the solid phase during the sorption process.

The cellulose-based material obtained with Soxhlet extraction using acetone as a solvent (SC-SOX-ACE) had good integrity and adsorption capacities, but was not as good as the material obtained with Soxhlet extraction using ethanol as a solvent (ND-SOX-ETH). The material obtained in the same conditions but with methanol as a solvent (NC-SOX-MET) did not have good structural and morphological characteristics; so, it was decided right from its synthesis–characterization phases not to test it for pollutant removal. However, for comparison purposes, its synthesis was investigated by LCA, as presented in the next section.

Adsorption tests with heavy metal ions (Cd^2+^ and Cr^2+^) were performed only with the material obtained by Soxhlet extraction with ethanol (NC-SOX-ETH). In total, 12 mg of adsorbent material was put in contact with 15 mL of mono-contaminated solutions at different concentrations and shaken for 24 h. The solution concentration ranged from 0.5 ppm to 200 ppm, to investigate the worst conditions of pollution. All materials were characterized chemically and morphologically to evaluate the success of the syntheses.

### 2.2. Life Cycle Assessment Methodology

#### 2.2.1. LCA Planning: System Limits, Functional Unit

Life cycle assessment was planned and performed considering the ISO 14040 and ISO 14044 standards [44]. The comparison of the environmental performances of the four materials obtained at lab scale to be used as sorbents in reactive capping for organic and inorganic pollutant removal from sea-water matrices and marine sediments was the major goal of this study. LCA was based on the laboratory-scale synthesis and initial testing of the 4 materials, as described in the previous section. The LCA study adopted a *cradle-to-gate* attributional approach considering the system boundaries as presented in Figure 1. Our analysis did not include any further processing nor any infrastructure processes related to the production of lab-scale equipment.

Two functional units were considered: 1 g of obtained nanocellulose bio-based material and 1 mg of eliminated pollutant to account for the material functionality.

The impact assessment method applied was ReCiPe 2016 Midpoint (H). The following 18 impact categories were considered: global warming, stratospheric ozone depletion, ionizing radiation, ozone formation human health, fine particulate matter formation, ozone formationterrestrial ecosystems, terrestrial acidification, freshwater eutrophication, marine eutrophication, terrestrial ecotoxicity, freshwater ecotoxicity, marine ecotoxicity, human carcinogenic toxicity, human non-carcinogenic toxicity, land use, mineral resource scarcity, fossil resource scarcity, and water consumption [45].

#### 2.2.2. Life Cycle Inventory

The inventory data is presented in Table 2. In processing the inventory, the following assumptions and limitations were employed. Cellulose microcrystalline powder was modelled based on literature data [46], considering only the processing operations. Electricity was modelled considering the Italian electricity mix for 2023, and electricity consumption was calculated based on the power rating of the laboratory equipment and operation time. This approach to electricity modeling led to an overestimation of electricity consumption, because the lab equipment was not optimized for these operations. The chemicals used in the syntheses were modelled by sourcing the corresponding data from EcoInvent 3.3. database.

For the LCA study, we used SimaPro LCA software (version 9.2.0.2), and all data were taken from the Ecoinvent database version 3. Most of the data from the Ecoinvent database were chosen using the methodological approach of attributional modelling, in which burdens are attributed proportionally to the specific processes (APOS, allocation at the point of substitution).

The data collected were then modelled using the ReCiPe Midpoint (H) (v1.02) method. The challenge with an LCA study based on lab-scale data regards the uncertainties, and it is very important to be precise with the input data quantities (e.g., amounts of chemicals and energy consumption).

## 3. Results

### 3.1. Environmental Profiles

As reported in the literature [28,47], the most used synthesis protocol to produce cellulose-based adsorbent materials is the freeze-drying process because it generates an aerogel-like material with a high surface-area-to-volume ratio. The goal of the LCA was to evaluate the environmental performance of this protocol and to compare it to the other tested syntheses routes which were based on Soxhlet extraction processes to remove the organic phase from the solid nanostructured modified cellulose sorbents.

**Figure 2:**
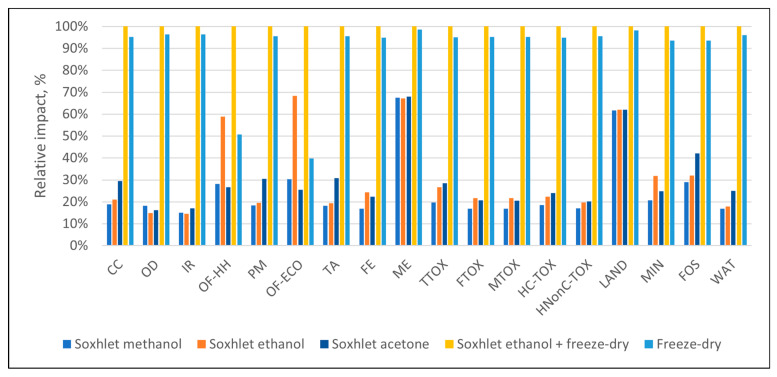
Impact comparison of synthesis methods per 1 g of synthesized materials (impact categories are abbreviated according to Table 3. Subsequent impact profiles use the same abbreviations).

Data in Table 3 present the values of the environmental impacts generated for the syntheses of 1 g of each sorbent material. As presented in Figure 2, the highest impacts in all categories appeared when the freeze-dry process was employed (per se or in combination with Soxhlet extraction), mainly due to the additional energy requirements, as discussed below. In general, the Soxhlet-only processes had impacts 31–85% lower than the freeze-dry processes. Considering that the freeze-dry alternative did not lead to structurally sound materials, this synthesis route was not considered for further analysis.

**Figure 3:**
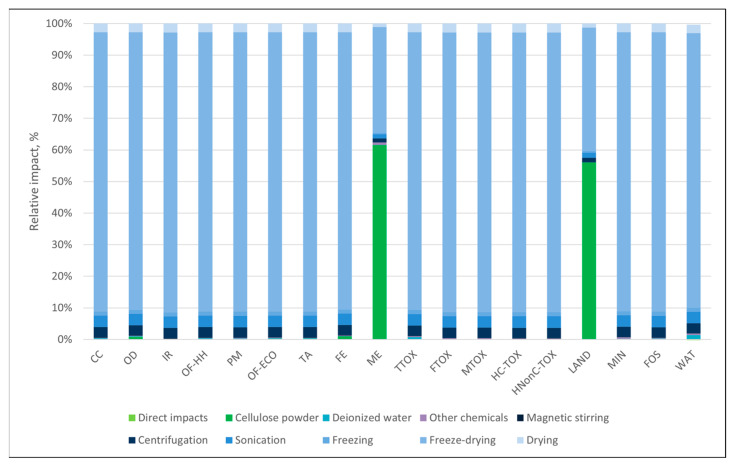
Cellulose-based materials obtained by the freeze-drying process.

**Figure 4:**
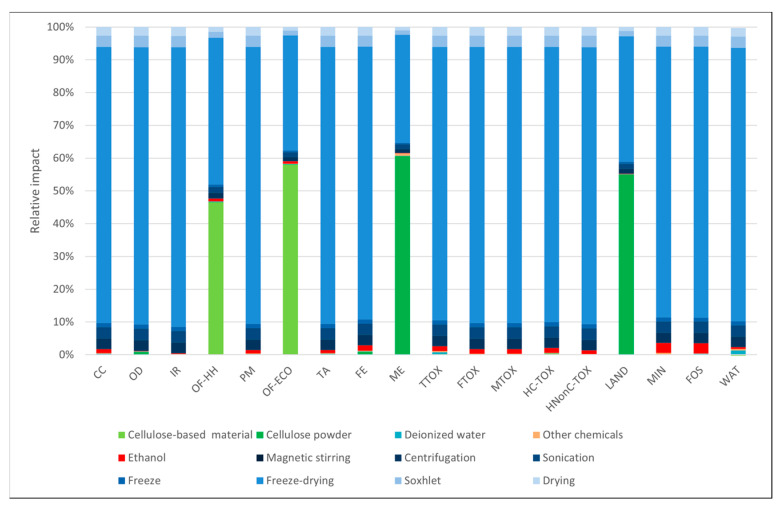
Cellulose-based materials obtained by the freeze-drying process and washing in the Soxhlet extractor using ethanol as a solvent.

In the case of the materials obtained through the freeze-dry process (Figure 3 and Figure 4), the environmental profiles were largely dominated by the contribution of electricity consumption, except for a few categories. In both cases, the electricity demand of the freeze-dry process was responsible for the largest part of the environmental impacts (82–85%) in most categories. For the cellulose-based freeze-dried (NC-FD) material (Figure 3), other significant contributions regarded the categories *marine eutrophication (ME)* and *land use (LAND)*, while in the case of the freeze-dry process coupled with Soxhlet extraction (NC-FD-SOX, Figure 4), the ethanol losses during the Soxhlet extraction process, which were measured at 10% of the initial ethanol volumes, contributed 46.6% and 58.1% of the total impact to the ozone formation categories (*OF-HH* and *OF-ECO*), respectively.

Although it was not tested for pollutant removal, in the initial environmental screening, the NC-SOX-MET material was considered because it was relevant to evaluate if the solvent choice had provided an important contribution to the overall impact.

**Figure 5:**
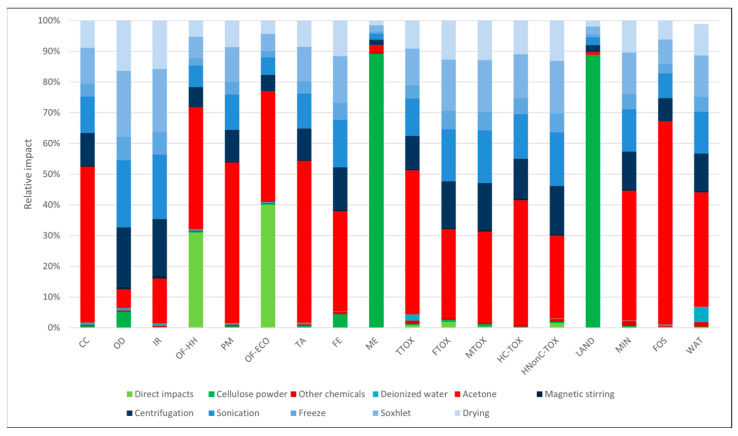
Cellulose-based materials obtained by Soxhlet extraction using acetone as a solvent.

**Figure 6:**
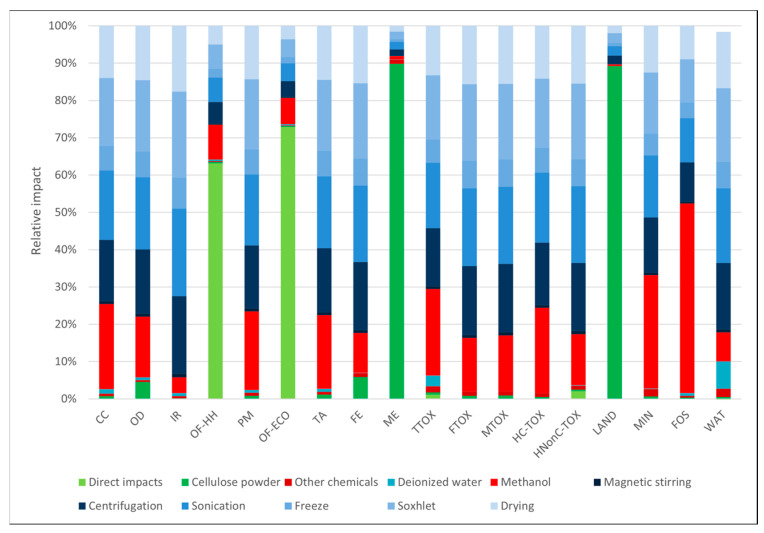
Cellulose-based materials obtained by Soxhlet extraction using methanol as a solvent.

**Figure 7:**
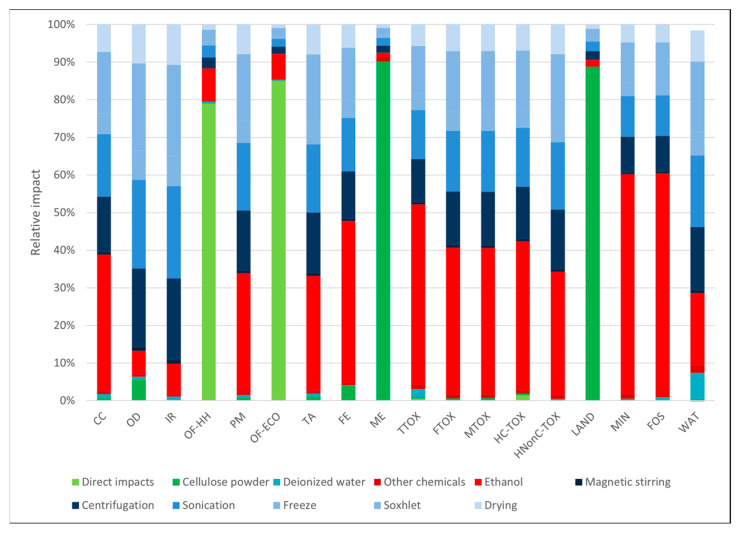
Cellulose-based materials obtained by Soxhlet extraction using ethanol as a solvent.

The choice of the extraction solvent was investigated, and the corresponding environmental profiles are presented in Figure 5, Figure 6 and Figure 7, where one may observe that the impact profile structure of the three options is similar, with a more balanced distribution between electricity consumption and the use of chemicals compared to the freeze-dry processes. Furthermore, data in Table 3 show that the impact differences among the three solvents lay between 4.84% and 41%, with two notable exceptions. Methanol seemed to have the lowest environmental impact in most categories, except the *ozone depletion* (OD) category, where it had a 10.90% and 17.6% higher impact than the other two options. A more balanced situation was observed for ethanol and acetone, with acetone having higher impacts in 10 impact categories, with great differences (from 37.3 to 41.4% greater), and ethanol generating higher impacts in only eight categories but with smaller differences (from 21.64 to 34.96%). A notable exception to this was *the ozone depletion category (OD)*, where ethanol emissions generated 52 to 62% higher impacts. All these differences stemmed from the different characterization factors of these pollutants in their respective categories.

### 3.2. Material Screening

These initial environmental profiles, together with the material testing data led to the screening of the cellulose-based materials, as well as to the definition of a second functional unit. This second functional considers the material functionality, as ISO 14040 recommends, and it was defined as 1 mg of sorbed pollutant.

After synthesis and characterization, three of the materials were used in adsorption tests of three organic dyes (4-nitrobenzaldehyde, 4-nitrophenol, and methylene blue), and the results are presented in Table 1 The cellulose-based material obtained with Soxhlet extraction using methanol as a solvent (NC-SOX-MET) was not tested because its characterization revealed a harder and more rigid structure compared to those of the other materials, and more important, it did not present the typical porous inner structure of the other materials enabling sorption.

The adsorption tests were implemented in a first phase with the study of the adsorption kinetics of three organic dyes. The first result that we were able to appreciate was the different material resistance during the 24 h of the tests, and this behavior had repercussions on the adsorption capacity of the adsorbent materials.

**Figure 8:**
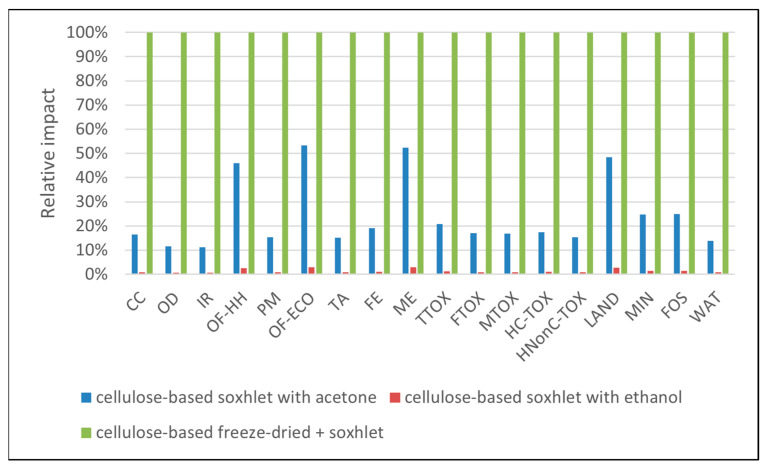
Environmental performance comparison for the removal of 1 mg of 4-nitrobenzaldehyde.

**Figure 9:**
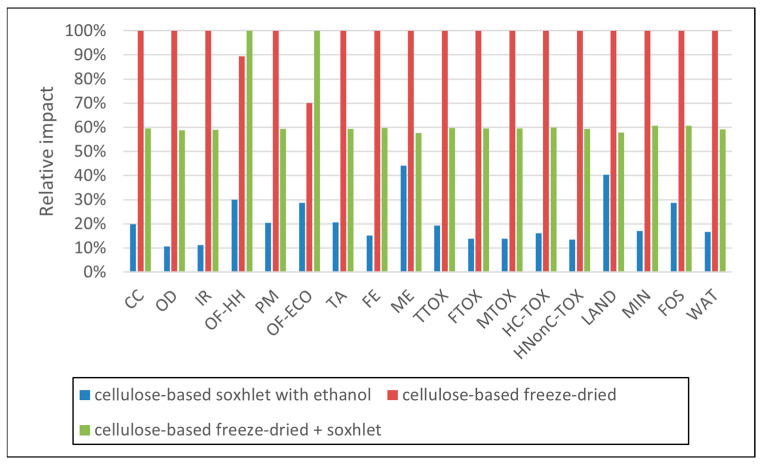
Environmental performance comparison for the removal of 1 mg of 4-nitrophenol.

**Figure 10:**
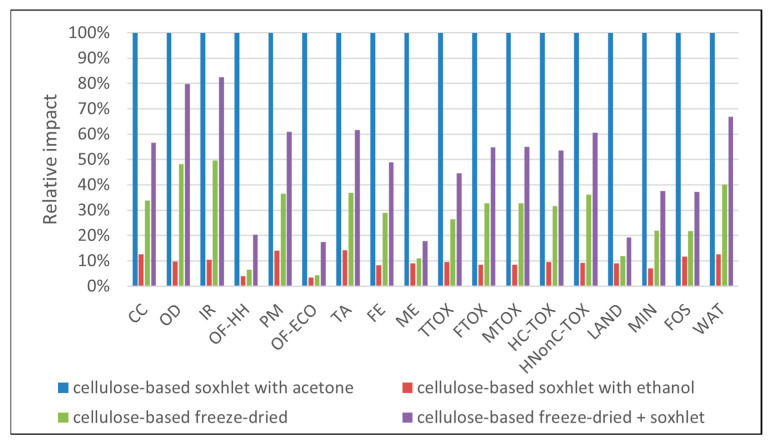
Environmental performance comparison for removal of 1 mg of methylene blue.

**Figure 11:**
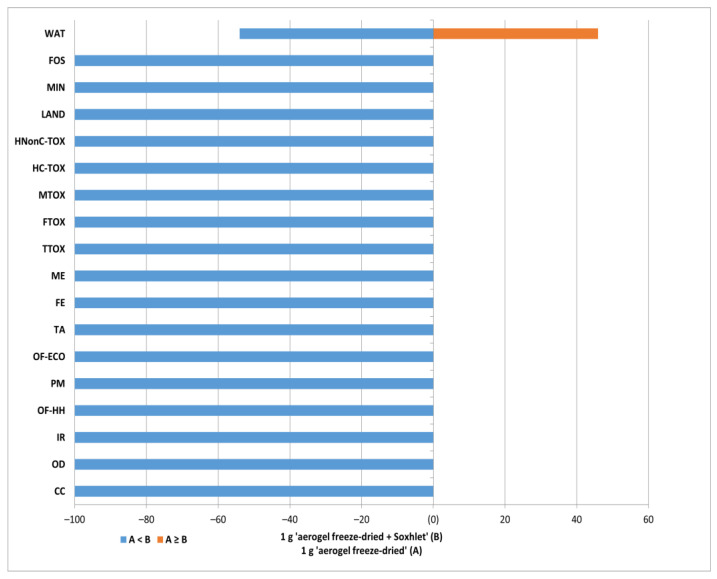
Monte Carlo analysis of NC-FD vs. NC-FD-SOX (probability of event A:NC-FD vs. probability of event B:NC-FD-SOX).

**Figure 12:**
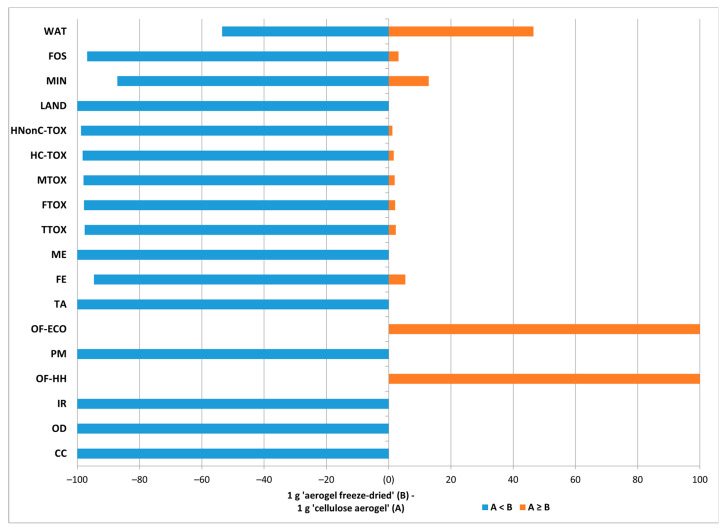
Monte Carlo analysis of NC-FD vs. NC-SOX-ETH (probability of event A:NC-FD-ETH vs. probability of event B:NC-FD).

The next step in the material screening process was to evaluate the environmental impacts by considering as a functional unit 1 mg of pollutant adsorbed. These environmental profiles are presented in Figure 8, Figure 9, Figure 10, Figure 11 and Figure 12. With respect to the organic pollutants, data in Figure 8, Figure 9 and Figure 10 show that, overall, the least environmental impact was generated by the material obtained with Soxhlet extraction using ethanol. The highest environmental impacts were generated by materials obtained by the freeze-dry process (NC-FD SOX for 4-nitrobenzaldehyde and NC-FD for 4 nitrophenol). For example, for the removal of 1 mg of 4-nitrobenzaldehyde, 5.81 kg eq CO_2_ were generated when using NC-FD-SOX, 0.95 kg eq CO_2_ when the sorbent was NC-SOX-ACE, and only 0.053 kg eq CO_2_ when using NC-SOX-ETH. The same patterns of considerably higher impacts were recorded in the toxicity related categories (3.90 kg eq 1,4-DCB when using NC-FD-SOX in TTOX, 0.81 kg eq 1,4-DCB when the sorbent was NC-SOX-ACE, and 0.045 kg eq 1,4-DCB when using NC-SOX-ETH) land use and fossil resources. For the removal of 1 mg of 4-nitrophenol, the highest impacts in all categories appeared when using NC-FD (for example, 6.75 kg eq CO_2_), followed by NC-FD-SOX (4.02 kg eq CO_2_) and NC-SOX-ETH with 1.34 kg eq. An exception was the ozone formation category, where due to the ethanol emissions, NC-FD-SOX generated higher impacts compared to the other materials. A rather peculiar situation regards the profiles obtained for the removal of 1 mg of methylene blue (Figure 10), where the highest impacts appeared when using the NC-SOX-ACE material, followed by NC-FD-SOX, NC-FD, and finally NC-SOX-ETH, with the least impacts in all categories. For comparison purposes, the recorded impacts in the climate change category were 0.9 kg for NC-SOX-ACE, which was the highest for the removal of this pollutant but much lower compared to the previous ones; 0.51 kg eq CO_2_ when using NC-FD-SOX; 0.30 kg eq CO_2_ when using NC-FD; and finally, 0.11 for NC-SOX-ETH. The same pattern of impact order was recorded for the other impact categories, and this was due to the smaller sorption capacity of NC-SOX-ACE for methylene blue compared to the other materials. One may notice that in these profiles, some of the materials were missing, i.e., NC-FD for the removal of 4-nitrobenzaldehyde, and NC-SOX-ACE for the removal of 4-nitrophenol, and this is because their adsorption test results were inconclusive.

These initial sorption tests on organic pollutants revealed that the cellulose-based material obtained via Soxhlet extraction using ethanol (NC-SOX ETH) was the most promising of all materials and so, it was also tested for the adsorption of two heavy metal ions (cadmium and chromium). The worst material was the cellulose-based freeze-dried material, followed by the cellulose-based freeze-dried material washed with the Soxhlet extractor. The cellulose-based material obtained with Soxhlet extraction using acetone as a solvent was not bad, but not better than the first two materials.

The results regarding the heavy metal ion sorption showed that the removal of cadmium ions had a 33% lower impact compared to the removal of chromium ions due to the higher sorption capacity of NC-SOX-ETH. In terms of absolute impact values, the removal of heavy metal ions led to considerably lower impacts compared to the removal of organic pollutants (e.g., 0.027 kg eq CO_2_ per 1 mg Cd^2+^ removed and 0.040 kg eq CO_2_ per 1 mg Cd^2+^ in the climate change category and 0.023 kg eq 1,4-DCB per 1 mg Cd^2+^ and 0.034 kg eq 1,4-DCB per 1 mg Cd^2+^).

### 3.3. Sensitivity Analysis

A sensitivity analysis was performed to understand and evaluate the stochastic uncertainties related to how the inventory data affected the life cycle impact results. This was performed by means of Monte Carlo simulations in 10,000 points, where multiple aspects were investigated. The structure of the impact profiles previously discussed showed that electricity was a major contributor to the overall impact profiles; so, it was expected that a change in these entries would cause variability in the results. This was confirmed by the data presented in Figure 11, where the likelihood of the NC-FD-SOX material having greater impacts than NC-FD is expressed as the difference between the cumulative probability distribution (on the *x*-axis), which means that the higher the percentage, the greater the probability for that situation and the smaller the associated uncertainty. Considering that the only difference between the two inventories was the supplementary electricity needed for Soxhlet extraction, the data in Figure 11 demonstrate that increasing the electricity use did not lead to changes in the uncertainties in all categories, except for the *water resources* category, where the close probabilities indicate high uncertainty.

For the Monte Carlo analysis of the cellulose-based material obtained by freeze-drying compared with the cellulose-based material obtained by Soxhlet extraction using ethanol as a solvent, we obtained confirmation of what was hypothesized in the previous paragraphs, namely, that the use of the Soxhlet extractor alone was mainly associated with energy consumption and that the impacts in the ozone depletion categories were greater in the case of NC-SOX-ETH due to the direct ethanol emissions.

## 4. Conclusions

This study focused on the comparison of the technical and environmental performances of four cellulose bio-based materials to be used in the decontamination of marine sediments. The main findings refer to the hierarchical categorization of the materials based on their technical and environmental performances in eliminating organic pollutants and heavy metal ions. The data coming from laboratory-scale experiments regarding the materials’ synthesis and testing were used as inputs in an LCA study that allowed to identify and quantify the types and values of environmental impacts associated with these materials.

From a technical point of view, it is possible to assert that among the materials obtained with the freeze-drying process, the one washed in the Soxhlet extractor (NC-FD-SOX) was better than the unwashed one (NC-FD). During the adsorption tests, it was possible to observe that the unwashed material was more fragile and gave worse results than the other material. When comparing the three materials obtained by only employing the Soxhlet extractor with different solvents (without freeze-dying), it was possible to observe that the material obtained with methanol was the worst as it did not have a good structure and was rigid and more compact than the others. The material obtained with acetone did not give good results in the adsorption tests, and the material obtained with ethanol was the best of these three.

If we refer to the environmental performance, LCA was used to compare the environmental impacts of the four cellulose-based materials synthesized at lab scale. In this respect, the cellulose-based material obtained via Soxhlet extraction with ethanol proved to be the best choice, as it had lower environmental impacts and proved to have the highest adsorption capacity for organic dyes and heavy metals. Using the freeze-dry process to produce cellulose-based adsorbents led to considerable higher impacts, mainly due to a much higher energy consumption. This study proved that LCA is a useful tool to optimize the sustainability of adsorbent materials alongside lab-scale experiments and confirms that the right direction to produce new performant and sustainable adsorbent materials involves not only choosing wastes as starting materials, but also optimizing the consumption of electricity used for the processes. It is also important to note that LCA analysis and environmental profiles are more appropriate when considering the material functionality by defining a proper functional unit.

Another important conclusion is that LCA is useful as an environmental performance screening instrument, especially when the technical criteria and testing data fail to provide results that will lead to clear and straightforward decisions regarding the suitability and sustainability of using these materials in pollutant sorption applications.

## Figures and Tables

**Table 1:** Adsorption capacity (expressed in mg/g) calculated by adsorption tests (4-nitrobenzaldehyde = NBA, 4-nitrophenol = NP, methylene blue = MB, cadmium = Cd^2+^, chromium = Cr^2+^).

Material Type	Maximum Sorption Capacity (mg/g)				
	NBA	NP	MB	Cd^2+^	Cr^2+^
Cellulose-based freeze-dried (NC-FD)	X	0.9	20	X	X
Cellulose-based freeze-dried, washed with Soxhlet extractor (NC-FD-SOX)	1.1	1.6	12	X	X
Cellulose-based, Soxhlet extraction with acetone (NC-SOX-ACE)	1.4	X	16	X	X
Cellulose-based, Soxhlet extraction with ethanol (NC-SOX-ETH)	1.4	1.5	25	38.9	42.3

Note: X—no results, the experiments did not give results because it was not possible to calculate the sorption capacity.

**Table 2:** Inventory for the cellulose materials.

Inventory Entry	Eco-Invent Process	Unit	Quantity for Cellulose-Based Material	Data Source/Comments
Inputs				
Cellulose microcrystalline powder	Cellulose powder	g	1.33	Modeled according to [46]
Potassium Bromide (KBr)	Bromine {GLO}|market for|APOS, U	g	0.20	
2,2,6,6-Tetramethylpiperidinyloxyl (TEMPO)	Dimethylamine (production from alcohols), at producer, 100% active substance/EU-27	g	0.03	Modeled as dimethylamine
Deionized water	water, deionized {Europe without Switzerland}|market for water, deionized|APOS, U	g	368.85	
NaClO 10%	Sodium hypochlorite/RER	g	15	
NaOH 0.5 M	Neutralizing agent, sodium hydroxide-equivalent {GLO}| market for|APOS, U	g	1.94	
HCl 37% *v*/*v*	Hydrochloric acid, without water, in 30% solution {RER}|market for|APOS, U	g	0.59	
bPEI Mw = 25,000	Ethylamine {RER}|production|APOS, U	g	1.36	
Ethanol	Ethanol, without water, in 99.7% solution, from ethylene {RER}| market for ethanol, without water, in 99.7% solution, from ethylene|APOS, U	g	394.5	
Magnetic stirring	Electricity, low voltage {IT}|electricity voltage transformation from medium to low voltage|APOS, U	KWh	0.02	
Centrifugation		KWh	0.425	
Sonication		KWh	0.48	
Freeze		KWh	0.17	
Soxhlet		KWh	0.468	
Drying process		KWh	0.21	
Outputs				
Ethanol	Ethanol, in air	g	40	
Chemically polluted water	Chemically polluted water	g	352.46	
Alcohols	Alcohols, unspecified	g	315	
Amine	Amine, tertiary	g	0.33	

**Table 3:** Environmental impacts values for obtaining 1 g of each material.

Impact Category	Unit	Abbrev.	Soxhlet Methanol	Soxhlet Ethanol	Soxhlet Acetone	Soxhlet Ethanol + Freeze-Dry	Freeze-Dry
Global warming	kg CO_2_ eq	CC	1.20 × 10^+0^	1.35 × 10^+0^	1.89 × 10^+0^	6.39 × 10^+0^	6.09 × 10^+0^
Stratospheric ozone depletion	kg CFC11 eq	OD	8.92 × 10^−7^	7.35 × 10^−7^	7.94 × 10^−7^	4.91 × 10^−6^	4.74 × 10^−6^
Ionizing radiation	kBq Co-60 eq	IR	1.05 × 10^−1^	1.01 × 10^−1^	1.18 × 10^−1^	6.94 × 10^−1^	6.69 × 10^−1^
Ozone formation, human health	kg NOx eq	OF-HH	5.96 × 10^−3^	1.24 × 10^−2^	5.62 × 10^−3^	2.11 × 10^−2^	1.07 × 10^−2^
Fine particulate matter formation	kg PM2.5 eq	PM	1.22 × 10^−3^	1.29 × 10^−3^	2.02 × 10^−3^	6.61 × 10^−3^	6.32 × 10^−3^
Ozone formation, terrestrial ecosystems	kg NOx eq	OF-ECO	8.34 × 10^−3^	1.87 × 10^−2^	7.00 × 10^−3^	2.74 × 10^−2^	1.09 × 10^−2^
Terrestrial acidification	kg SO_2_ eq	TA	3.57 × 10^−3^	3.80 × 10^−3^	6.06 × 10^−3^	1.96 × 10^−2^	1.88 × 10^−2^
Freshwater eutrophication	kg P eq	FE	3.08 × 10^−4^	4.46 × 10^−4^	4.09 × 10^−4^	1.83 × 10^−3^	1.73 × 10^−3^
Marine eutrophication	kg N eq	ME	2.90 × 10^−4^	2.89 × 10^−4^	2.92 × 10^−4^	4.30 × 10^−4^	4.23 × 10^−4^
Terrestrial ecotoxicity	kg 1,4-DCB	TTOX	8.49 × 10^−1^	1.15 × 10^+0^	1.23 × 10^+0^	4.30 × 10^+0^	4.08 × 10^+0^
Freshwater ecotoxicity	kg 1,4-DCB	FTOX	2.85 × 10^−2^	3.68 × 10^−2^	3.50 × 10^−2^	1.69 × 10^−1^	1.61 × 10^−1^
Marine ecotoxicity	kg 1,4-DCB	MTOX	3.74 × 10^−2^	4.79 × 10^−2^	4.52 × 10^−2^	2.21 × 10^−1^	2.10 × 10^−1^
Human carcinogenic toxicity	kg 1,4-DCB	HC-TOX	4.57 × 10^−2^	5.49 × 10^−2^	5.90 × 10^−2^	2.46 × 10^−1^	2.33 × 10^−1^
Human non-carcinogenic toxicity	kg 1,4-DCB	HNonC-TOX	6.01 × 10^−1^	6.93 × 10^−1^	7.10 × 10^−1^	3.52 × 10^+0^	3.36 × 10^+0^
Land use	m^2^a crop eq	LAND	1.91 × 10^+0^	1.92 × 10^+0^	1.92 × 10^+0^	3.09 × 10^+0^	3.04 × 10^+0^
Mineral resource scarcity	kg Cu eq	MIN	1.22 × 10^−3^	1.87 × 10^−3^	1.46 × 10^−3^	5.88 × 10^−3^	5.51 × 10^−3^
Fossil resource scarcity	kg oil eq	FOS	5.87 × 10^−1^	6.47 × 10^−1^	8.50 × 10^−1^	2.02 × 10^+0^	1.89 × 10^+0^
Water consumption	m^3^	WAT	2.08 × 10^−2^	2.21 × 10^−2^	3.09 × 10^−2^	1.24 × 10^−1^	1.19 × 10^−1^

## Data Availability

The original contributions presented in the study are included in the article. The data presented in this study are available on request from the first or corresponding authors.
